# Insect CncC: Roles in Redox Homeostasis, Physiology, and Insecticide Resistance, with Prospects for Biotechnological Applications

**DOI:** 10.3390/insects17101061

**Published:** 2026-10-14

**Authors:** Yongjie Cen, Yumei Chen, Ke Wang, Dasong Chen, Shakil Ahmad, Momana Jamil, Gecheng Ouyang, Xiang Meng

**Affiliations:** 1 Guangdong Key Laboratory of Animal Conservation and Resource Utilization, Institute of Zoology, Guangdong Academy of Sciences, Guangzhou 510275, China; chendasong@gmail.com (D.C.); mshakil@aup.edu.pk (S.A.); 18922369378@189.cn (G.O.); 2 Precise Genome Engineering Center, School of Life Sciences, Guangzhou University, Guangzhou 510006, China; 2022010195@m.scnu.edu.cn; 3 Guangdong Provincial Key Laboratory of Biotechnology for Plant Development, School of Life Sciences, South China Normal University, Guangzhou 510631, China; 19878582790@163.com; 4 School of Ecology, Hainan University, Haikou 570228, China; jamilmomana@gmail.com

**Keywords:** CncC, Nrf2, insects, oxidative stress, detoxification metabolism, growth and development, insecticide resistance

## Abstract

The Cap ‘n’ collar isoform C (CncC) transcription factor, the insect ortholog of mammalian nuclear factor erythroid 2-related factor 2 (Nrf2), serves as a central regulator of redox homeostasis and xenobiotic detoxification. Increasing evidence demonstrates that the functions of CncC extend beyond stress responses to include metamorphosis, immunity, reproduction, aging, and the development of metabolic insecticide resistance. In this review, we synthesize recent advances in insect CncC biology, with a focus on the conserved CncC/Keap1-ARE signaling pathway and its role in the detoxification of phytochemicals and insecticides. We further discuss insect-specific regulatory interactions between CncC and ecdysone signaling, as well as the gut microbiota, and compare insect CncC with mammalian Nrf2. From an applied perspective, we discuss CncC as a target for resistance management, including strategies involving RNAi and small-molecule inhibitors, its potential application in inducible bioreactors, and its value as a biomarker in environmental risk assessment. We conclude by identifying key open questions, including tissue-specific regulation, fitness costs of constitutive activation, and field relevance of CncC-mediated resistance, and highlight how addressing these gaps can support the development of sustainable pest control strategies.

## Simple Summary

Insects are constantly exposed to toxic chemicals from their diet and environment. A protein called CncC acts as a master switch that activates protective genes, helping insects detoxify harmful substances and resist oxidative damage. Recent research has shown that CncC also performs other important functions: it helps regulate insect growth and metamorphosis, supports immune defense, influences reproduction and aging, and can contribute to insecticide resistance in pests. This review consolidates the current knowledge of CncC across different insect species. We explain how CncC is activated, which genes it controls, and how it interacts with hormones and gut microbes. We also discuss how this knowledge can be applied in practical ways, such as improving pest control, developing inducible bioreactors, and monitoring environmental pollution. Finally, we highlight important unanswered questions. A deeper understanding of CncC may help scientists design safer and more sustainable strategies for managing insect pests while protecting beneficial insects and the environment.

## 1. Introduction

Living organisms are constantly threatened by oxidative stress arising from endogenous metabolic byproducts and exogenous noxious agents. In response to this challenge, evolution has endowed eukaryotic systems with an elaborate repertoire of cytoprotective defenses, chief among them the transcriptional mobilization of genes encoding detoxification and antioxidant enzymes. In mammals, the transcription factor nuclear factor erythroid 2-related factor 2 (Nrf2) is a key regulator of this defense system [1,2]. Its insect ortholog, Cap ‘n’ collar isoform C (CncC), likewise belongs to the Cap ‘n’ Collar (Cnc) family of basic leucine zipper (bZIP) proteins and occupies a position of comparable importance in the maintenance of cellular redox equilibrium, xenobiotic metabolism, and the regulation of various physiological processes [3,4].

The advent of high-throughput sequencing, gene editing, and proteomics technologies has led to significant advances in insect CncC research. Investigations have expanded beyond validating its classical detoxification function to reveal its roles in insect metamorphosis, reproduction, aging, immune defense, and insecticide resistance [5,6,7,8,9]. Of particular note, the crosstalk linking CncC activity to the ecdysone signaling pathway and the gut microbiome constitutes an insect-specific regulatory architecture [10,11]. In this review, we provide a systematic synthesis of recent advances, compare the similarities and differences between CncC and its mammalian counterpart Nrf2, and explore the translational prospects of targeting the CncC pathway for pest management.

## 2. Discovery and Major Functions of Nrf2 in Mammals

### 2.1. Discovery and Structural Features of the CncC Homologous Protein Nrf2

Nrf2 was first discovered in 1994 by Yuet Wai Kan’s laboratory during studies on globin gene regulation. They identified a nuclear protein that bound with high affinity to the β-globin locus control region and named it Nrf2 due to its homology with the erythroid transcription factor NF-E2 [12]. Nrf2 belongs to the Cnc transcription factor family and contains a highly conserved bZIP domain responsible for binding to the antioxidant response element (ARE) on DNA and initiating the transcription of downstream target genes [13,14].

**Figure 1:**
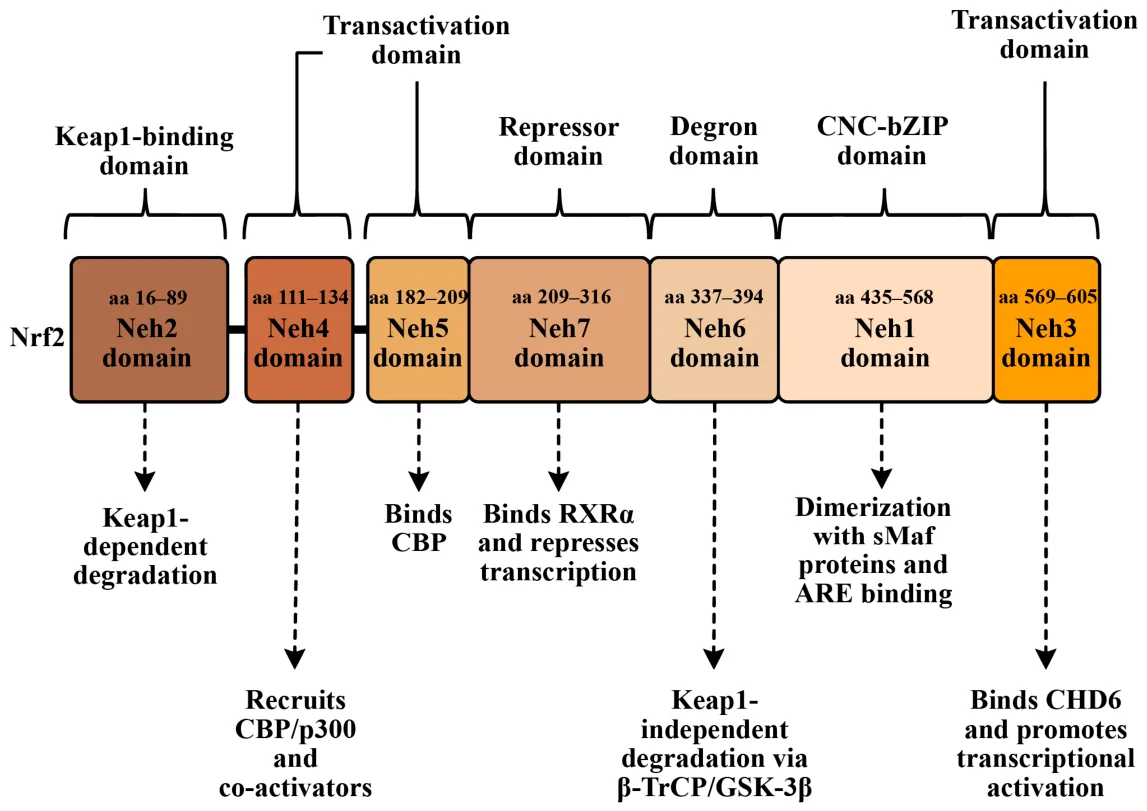
Protein domains of the CncC homologous protein Nrf2 and the functions of different domains. Nrf2 protein contains seven highly conserved Neh domains. The Neh1 domain contains the bZIP motif, which is responsible for dimerization with sMaf proteins and binding to the antioxidant response element (ARE). The Neh2 domain serves as the Keap1-binding domain and mediates Keap1-dependent ubiquitination and degradation. The Neh3 domain binds the transcriptional coactivator CHD6 and promotes transcriptional activation. The Neh4 and Neh5 are both transactivation domains that promote transcriptional activation by recruiting CBP/p300. The Neh6 domain functions as a degron domain and mediates Keap1-independent degradation via the β-TrCP/GSK-3β pathway. The Neh7 domain binds RXRα and represses transcription. CBP: CREB (cAMP responsive element binding protein) binding protein; RXRα: retinoic X receptor alpha; GSK-3β: glycogen synthase kinase-3β; CHD6: a chromo-ATPase/helicase DNA binding protein.

Structurally, Nrf2 contains seven Neh (Nrf2-ECH homology) domains [15] (Figure 1). The Neh1 domain contains the bZIP region required for DNA binding and dimerization with small Maf proteins (musculoaponeurotic fibrosarcoma) [16]. The Neh2 domain binds to the negative regulator Keap1 (Kelch-like ECH-associated protein 1), mediating Nrf2 ubiquitination and subsequent proteasomal degradation [17]. The Neh3, Neh4, and Neh5 domains participate in the recruitment of transcriptional coactivators [18,19], and the Neh6 domain is involved in the Keap1-independent degradation pathway [20].

### 2.2. Core Functions of the CncC Homologous Protein Nrf2: Antioxidant Defense and Detoxification

Nrf2 is widely regarded as the “master switch” of the cellular antioxidant defense system. Under physiological (unstressed) conditions, Nrf2 forms a complex with Keap1 in the cytoplasm. Keap1 acts as a substrate adaptor protein for the Cullin 3 (Cul3)-based ubiquitin E3 ligase, promoting Nrf2 ubiquitination and rapid degradation by the proteasome, thereby maintaining Nrf2 at a low basal level [21,22]. When cells are exposed to electrophiles, oxidative stress, or xenobiotic chemicals, critical cysteine residues of Keap1 are modified, leading to conformational changes that disrupt Nrf2 ubiquitination. Consequently, newly synthesized Nrf2 is stabilized and translocates into the nucleus [23,24]. Once in the nucleus, Nrf2 forms heterodimers with small Maf proteins, recognizes and binds to ARE in the promoter regions of target genes, and activates the expression of phase II detoxification and antioxidant enzyme genes, including glutathione S transferases (GSTs), UDP-glucuronosyltransferases (UGTs), NAD(P)H: quinone oxidoreductase 1 (*NQO1*), heme oxygenase-1 (*HO-1*), and superoxide dismutase (*SOD*) [25,26,27].

### 2.3. Other Functions of the CncC Homologous Protein Nrf2

**Figure 2:**
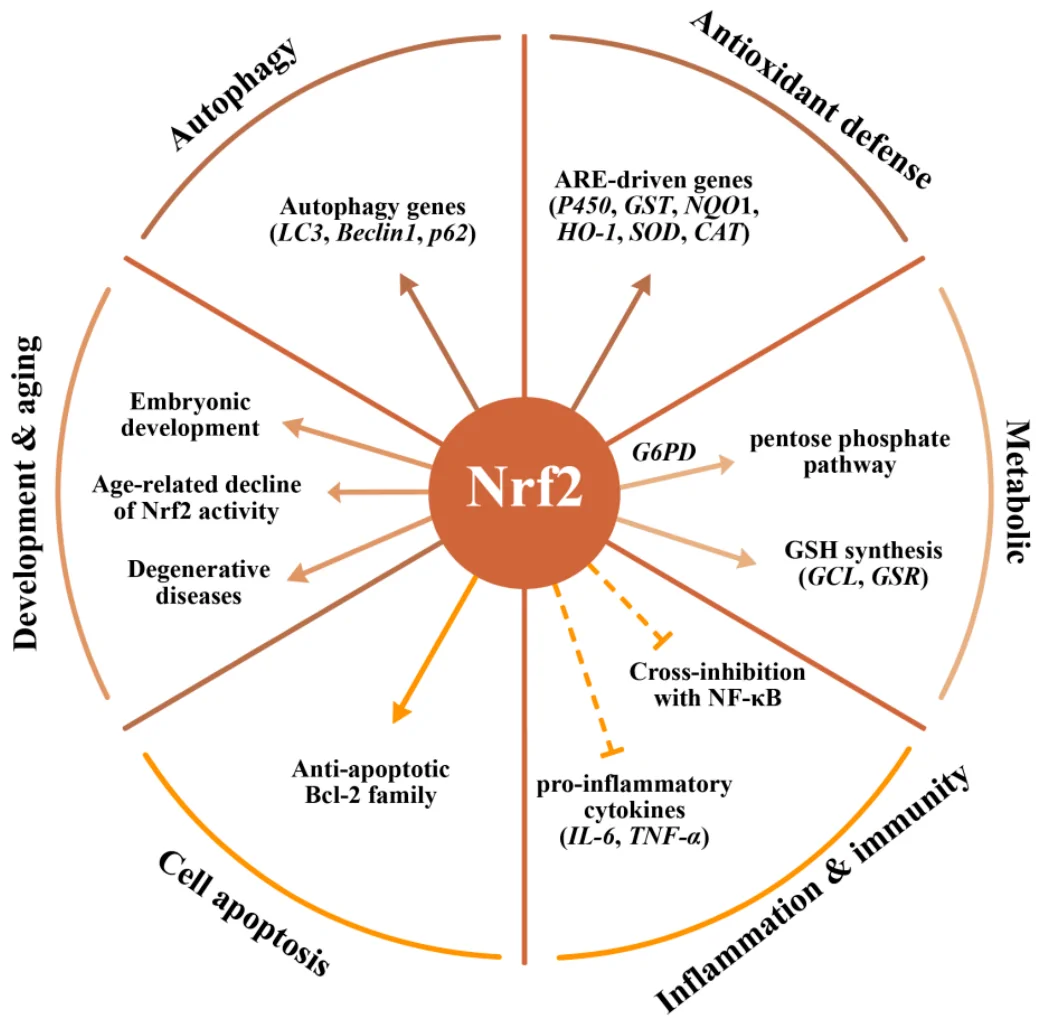
The core biological function of the CncC homologous protein Nrf2. Nrf2 participates in cellular antioxidant defense by regulating genes such as *GST*, *NQO1*, and *SOD*. Nrf2 is involved in cellular metabolism through the regulation of the pentose phosphate pathway and the GSH synthesis pathway. Nrf2 cross-talks with the NF-κB signaling pathway and suppresses pro-inflammatory cytokines including *IL-6* and *TNF-α*, thereby exerting anti-inflammatory effects. Nrf2 exerts anti-apoptotic effects by upregulating the expression of anti-apoptotic genes (such as members of the Bcl-2 family). Nrf2 regulates the expression of autophagy-related genes such as *p62*, *LC3*, and *Beclin1*, thereby promoting autophagosome formation. Nrf2 also participates in the regulation of embryonic development and the aging process in organisms. *LC3*: Microtubule-associated protein 1A/1B-light chain 3; *GST*: glutathione-S-transferase; *NQO1*: NAD(P)H:quinone oxidoreductase-1; *HO-1*: heme oxygenase-1; *SOD*: superoxide dismutase; *CAT*: catalase; *G6PD*: glucose-6-phosphate dehydrogenase; GSH: glutathione; *GCL*: glutamate-cysteine ligase; *GSR*: glutathione reductase; *IL-6*: interleukin-6; *TNF-α*: tumor necrosis factor alpha; Bcl-2: B-cell lymphoma protein 2. Arrow head: activation; Bar head: inhibiton; Solid line: direct effect; Dashed line: indirect effect.

Beyond its well-established role in antioxidant defense, Nrf2 is increasingly recognized for its involvement in a wide array of physiological and pathological processes (Figure 2). Nrf2 exerts a broad influence over cellular metabolism and redox status, partly through transcriptional control of pentose phosphate pathway enzymes, with glucose-6-phosphate dehydrogenase (*G6PD*) being a notable example [28,29]. An additional layer to its regulatory repertoire involves a mutual antagonism with NF-κB signaling; this cross-inhibitory crosstalk dampens the production of pro-inflammatory cytokines, thereby conferring anti-inflammatory benefits [30,31]. Its protective functions extend to apoptosis inhibition via upregulation of anti-apoptotic factors such as Bcl-2 [32], while in the context of malignancy, persistent Nrf2 activation is a recognized driver of chemoresistance [33]. The age-related erosion of Nrf2 activity has also been firmly implicated in the progression of degenerative diseases [34], contrasting with its essential developmental role in promoting the maturation of vital organs [35]. Collectively, the depth of insight gleaned from mammalian Nrf2 studies has established a comprehensive understanding of its function, ranging from molecular mechanisms to therapeutic applications, providing a valuable framework for dissecting the functions of its insect ortholog, CncC.

## 3. Discovery and Major Functions of CncC in Insects

### 3.1. Discovery and Evolutionary Conservation of CncC

**Figure 3:**
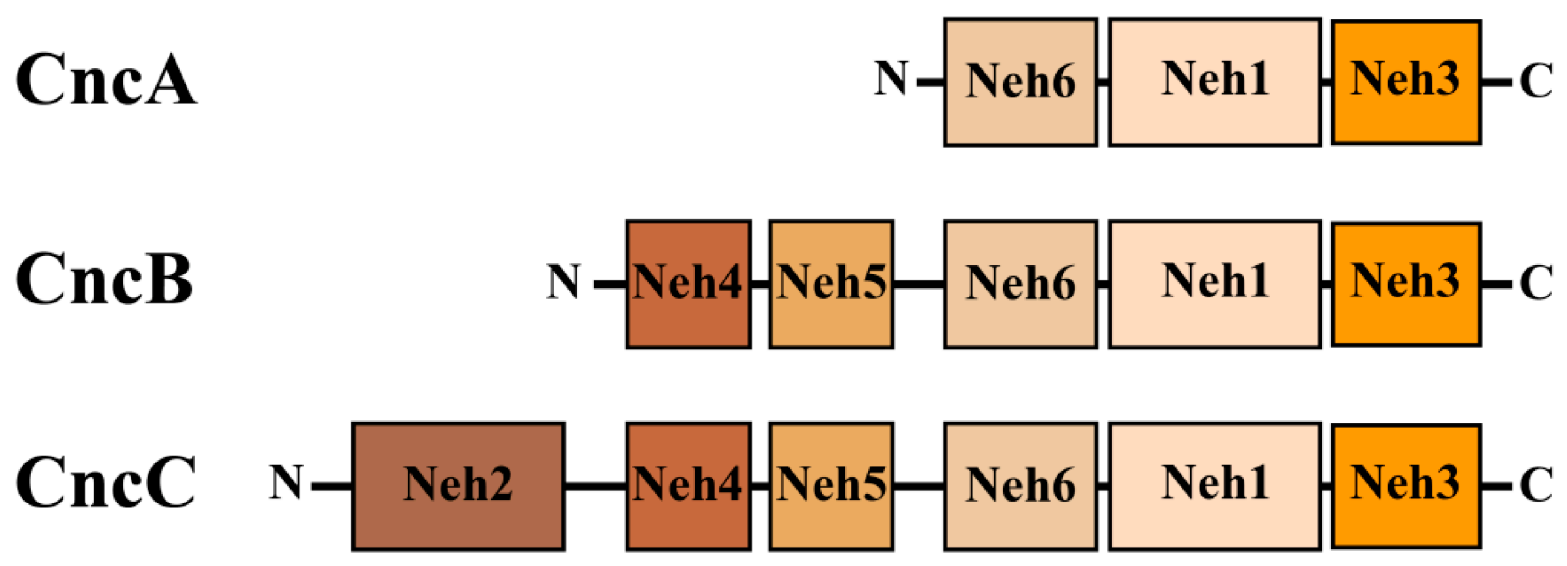
Protein domains of three Cnc isoforms (CncA, CncB and CncC) in insects. The NEH2 domain mediates interaction with Keap1. NEH3, NEH4, and NEH5 function as transcriptional activation domains. The NEH1 domain is critically involved in the process of DNA binding.

The insect transcription factor CncC was initially identified in *Drosophila melanogaster* as a constituent of the Cnc family [36]. It belongs to a broader gene set that includes isoforms like CncA and CncB, whose primary divergence lies within their NH_2_-terminal sequences (Figure 3) [37]. Homology-based comparative analyses have identified CncC as the ortholog of mammalian Nrf2, and the protein product encoded by this gene retains Neh domains consistent with those found in mammals, spanning Neh1 to Neh6 (Figure 3) [37,38,39]. Consistent with observations in mammalian systems, the negative regulation of CncC mediated by Keap1, as well as the formation of CncC-Maf dimers that mediate transcriptional regulation of downstream target genes, has been experimentally validated in insect species [40,41]. Nonetheless, whether all structural and regulatory characteristics of this signaling axis are completely evolutionarily conserved remains to be fully elucidated. Compared with CncC, the current functional research on CncA and CncC is still extremely limited, and their specific biological functions in insect physiological processes remain largely uncharacterized.

Evolutionary analyses indicate that the CncC/Nrf2 system is highly conserved across a broad evolutionary spectrum, from invertebrates to vertebrates [42]. In addition to *Drosophila*, CncC orthologs have been identified in various important insect orders, including Lepidoptera (*Bombyx mori*, *Spodoptera frugiperda*, and *Helicoverpa armigera*) and Coleoptera (*Tribolium castaneum*), and their core functions have been demonstrated to be mediated by the conserved “CncC-Keap1-ARE” signaling axis [43,44,45,46].

### 3.2. Core Functions of CncC: Antioxidant Defense and Detoxification Metabolism

#### 3.2.1. Overview of the CncC-Keap1-ARE Pathway in Insects

Similar to mammalian Nrf2, CncC serves as a core transcription factor in insects in response to oxidative stress and xenobiotic toxicity [47,48,49]. Classical research models are primarily based on *D. melanogaster* and *B. mori*. For example, in *Drosophila*, RNAi-mediated knockdown of *CncC* gene significantly reduces larval tolerance to exogenous toxic compounds such as phenobarbital, accompanied by downregulation of genes encoding detoxification enzymes such as P450s and GSTs [50]. Conversely, overexpression of *CncC* enhances antioxidant capacity [51].

A critical conceptual distinction derived from entomological investigations is the separation between inducible activation and constitutive activation of the CncC signaling pathway. Inducible activation constitutes the canonical adaptive response, in which transient exposure to stress stimuli triggers post-translational modification of Keap1, which subsequently facilitates CncC protein stabilization, nuclear translocation, and transcriptional upregulation of cytoprotective target genes. Once the stress stimulus is removed, pathway activity is restored to baseline levels [52]. In comparison, constitutive activation results from genetic or epigenetic modifications that dissociate CncC from its negative regulator Keap1, which drives sustained overexpression of downstream detoxification genes even when no inducing stimulus is present [47]. This distinction is fundamental to understanding both the ecological dominance of polyphagous herbivorous insects and the evolutionary emergence of insecticide resistance. In the context of insecticide resistance, constitutive activation can originate from mutations occurring within the CncC coding region itself, structural or regulatory alterations of Keap1, or modifications of upstream regulatory factors that act on the CncC pathway. However, the exact genetic architecture underlying this phenotype remains poorly resolved in most pest species [53].

It should be emphasized that the majority of the canonical Keap1-Nrf2 activation pathway model was initially established in mammalian research systems. In insects, the negative regulation of CncC by Keap1 and the binding of CncC-Maf dimers to antioxidant response elements (AREs) have been experimentally verified in *D. melanogaster* [40,41,54]. Nevertheless, direct experimental evidence supporting the cysteine-mediated sensing function of Keap1 and Keap1-Cul3-dependent ubiquitination and turnover of CncC in insects is still insufficient, and current understandings of these processes are primarily extrapolated from findings in mammalian studies [37,50].

#### 3.2.2. CncC in the Detoxification of Phytochemicals

In herbivorous insects, CncC assumes a particularly prominent role in the detoxification of phytochemicals [52,55,56]. This is exemplified in lepidopteran pests, where dietary exposure to such compounds triggers rapid activation and nuclear translocation of midgut CncC, which in turn drives the expression of a broad suite of detoxification enzymes, including multiple cytochrome P450s, GSTs, and UGTs, thereby establishing a wide-spectrum xenobiotic metabolic network [57].

In *H. armigera*, tolerance to flavonoids has been linked to CncC-dependent regulation of *CYP321A1*, a cytochrome P450 capable of metabolizing a diverse array of phytochemicals [58]. Adaptation to xanthotoxin in *Spodoptera litura* similarly hinges on CncC-mediated upregulation of UGT genes [59], and CncC is known to orchestrate the detoxification of flavonoids, xanthotoxin, and curcumin in this species through coordinated control of CYP321A subfamily gene expression [60]. In *Micromelalopha troglodyta*, CncC confers tolerance to tannic acid and quercetin through direct transcriptional activation of *GSTt1* [61]. Beyond the order Lepidoptera, existing research has demonstrated that CncC orthologs contribute to the adaptation of coleopteran and hemipteran herbivores to host plant defenses, suggesting that this regulatory axis constitutes an evolutionarily conserved strategy for phytochemical stress tolerance [55,62]. Furthermore, the co-option of CncC signaling by specialist herbivores to tolerate high concentrations of specific host plant toxins constitutes a typical example of molecular adaptation during plant–insect coevolutionary arms races [63]. In this context, CncC acts as an integrator that couples the perception of phytochemical stress to the deployment of appropriate detoxification responses, which ultimately enables herbivores to utilize chemically defended host plants [64]. Although extant research has predominantly centered on phytophagous pests, whether CncC executes comparable functional roles in hematophagous insects and vectors of veterinary and medical importance remains an unresolved yet significant scientific question.

#### 3.2.3. CncC in Metabolic Resistance to Insecticide

Beyond phytochemicals, the ability of CncC to regulate the expression of detoxification enzymes establishes a critical molecular foundation for the development of metabolic resistance to synthetic insecticides in agricultural pests and disease vectors [4,65,66,67]. Accumulating evidence from studies covering multiple insect orders has indicated that constitutive or inducible CncC activation acts as a core driver for the formation of insect resistance phenotypes. One of the earliest and most influential empirical validations of this principle demonstrated that resistant strains of *D. melanogaster* display constitutive activation of the Nrf2/CncC pathway, which subsequently upregulates a cohort of detoxification genes, ultimately conferring broad-spectrum resistance [47]. This principle has subsequently been corroborated across a variety of pest species. In the Colorado potato beetle (*Leptinotarsa decemlineata*), CncC directly regulates the cytochrome P450s and ABC transporters that underpin imidacloprid resistance [68,69]. In *S. litura*, CncC activation mediates resistance to lambda-cyhalothrin and indoxacarb via the coordinated upregulation of *CYP6AB12*, carboxylesterase *CXE1* and the ABC transporter *ABCH-1* [70,71]. Analogously, in *Nilaparvata lugens*, CncC-mediated induction of UGT and P450 gene clusters is significantly correlated with imidacloprid resistance [72,73]. Considering that metabolic resistance is also a core concern in hematophagous insects and veterinary vectors, extending CncC-related research to these species will provide important value for the interpretation and management of insecticide resistance across a broader range of pest species. In *Aedes albopictus*, mutations in *AalND4* can induce mitochondrial reactive oxygen species bursts that activate the CncC signaling pathway, while silencing *AalCncC* can restore sensitivity to dimefluthrin via downregulating P450s, GSTs and ABCs [74]. Taken together, the above findings support the view that the CncC-Keap1 pathway may act as an important regulatory node for metabolic insecticide resistance, although its relative contribution varies among species and populations and remains to be rigorously quantified under field conditions. Instead of accumulating independent resistance mutations in individual detoxification genes, insect populations can acquire broad-spectrum resistance via genetic alterations that lead to the constitutive activation of this single regulatory pathway, which simultaneously drives the upregulated expression of dozens of detoxification-related protective genes [9,47].

To facilitate comparison and explicitly clarify the strength of supporting evidence, representative studies of CncC-mediated detoxification in insects have been summarized in Table 1. It should be emphasized that alterations in CncC expression alone are insufficient to confirm pathway activation, and the altered expression of downstream genes induced by CncC manipulation does not inherently provide definitive proof of direct transcriptional regulation. Accordingly, Table 1 explicitly distinguishes among three categories of evidence: direct molecular evidence (e.g., ChIP, EMSA, reporter assays), functional genetic evidence (e.g., RNAi, Agonist), and expression correlation evidence.

### 3.3. Other Functions of CncC in Insects

Compared with mammalian Nrf2, CncC exhibits unique biological functions in insects, which are often closely associated with insect-specific life cycles, metamorphosis, and ecological adaptability.

#### 3.3.1. Synergistic Regulation of Insect Metamorphosis with Hormonal Signals

An intriguing finding is the close crosstalk between CncC and the ecdysone (20-hydroxyecdysone, 20E) signaling pathway [79,80]. Studies have shown that 20E induces the expression of CncC, and in turn, CncC participates in the regulation of metamorphosis-related gene expression [6,81]. In *Drosophila*, loss of CncC function leads to reduced ecdysone levels in larvae and delays pupariation [10,82]. Consistently, in the mosquito *Aedes aegypti*, CncC forms a heterodimer with the 20E-induced bZIP transcription factor MafB, which functions to maintain a high titer of 20E and activate the expression of *vitellogenin* following blood meal ingestion [6]. Furthermore, CncC is also involved in regulating juvenile hormone (JH) levels. In *T. castaneum*, CncC downregulates JH titers by promoting the expression of juvenile hormone-degrading enzymes [83]; when *CncC* is knocked down, JH levels increase, resulting in failure of larval-pupal transition [84]. These findings indicate that there exists a bidirectional regulatory association between CncC and hormonal signaling, yet the causal direction of this association and whether the observed effects are mediated by direct or indirect interactions remain to be fully elucidated. Specifically, it remains largely ambiguous whether the developmental phenotypes detected following CncC manipulation are direct outcomes of perturbed hormonal signaling, or secondary effects caused by changes in detoxification processes, nutritional status, or redox homeostasis. Furthermore, the majority of current evidence is derived from studies on *D. melanogaster* and *T. castaneum*; thus the conservation of this regulatory relationship across other insect orders requires empirical verification rather than being taken for granted. In summary, the findings presented to date indicate that CncC is not merely a stress-responsive factor but also a critical node that integrates metabolic status and developmental progression. This mechanism, which couples nutritional and metabolic stress with developmental signals, may represent an evolutionary strategy for insects to adapt to complex and fluctuating environments.

#### 3.3.2. Gut Homeostasis and Microbial Interactions

**Figure 4:**
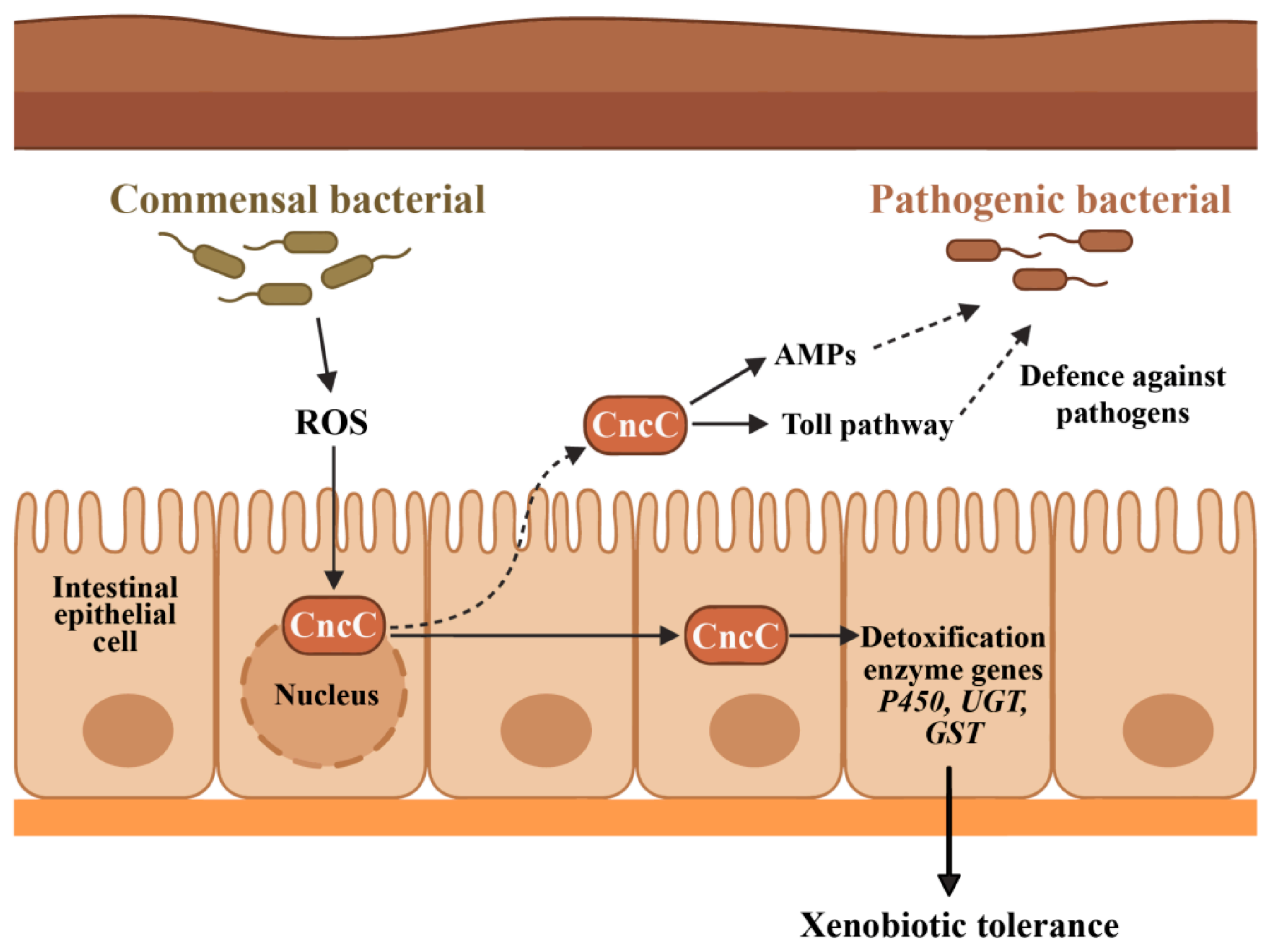
A model illustrating the function of CncC in gut homeostasis and microbe interaction in *Drosophila*. Gut commensal microbes regulate the activity of CncC in intestinal epithelial cells through the production of reactive oxygen species (ROS). Activated CncC drives the expression of a series of downstream detoxification enzymes (such as *P450* and *GST*), thereby enhancing insect tolerance to environmental xenobiotics. In addition, CncC participates in regulating the expression of antimicrobial peptides and intestinal immune responses, thereby influencing the homeostasis of commensal microbiota and the clearance of pathogenic bacteria. AMPs: antimicrobial peptides. Arrow head: activation; Solid line: direct effect; Dashed line: indirect effect.

The insect gut constitutes the primary interface for encounters with both xenobiotics and microbial pathogens. CncC plays a distinctive role in preserving gut homeostasis [85,86,87,88] (Figure 4). Work in *Drosophila* has recently demonstrated that CncC not only drives antimicrobial peptide expression but also engages cooperatively with the Toll immune pathway to defend against enteric pathogens [89,90,91]. Adding a further layer of complexity, gut commensal microbiota can modulate CncC activity through the release of metabolites (such as reactive oxygen species), thereby indirectly shaping host detoxification capacity and immune status [11,92,93], although it is often unclear whether these ROS are produced directly by microbes or induced in host cells, and whether other indirect effects on host redox status are involved. This “microbiota-host” regulatory axis impinging on CncC has been reported in mammals as well [94,95]; in insects, the consequences may be more immediate in some systems, potentially influencing adaptation to dietary xenobiotics and interaction with pathogen vectors such as mosquitoes [92,96]. At present, the available evidence supporting the microbiota–host–CncC axis is still predominantly correlative, and heterogeneity exists across different species and experimental systems. Furthermore, few studies have integrated microbiota depletion, germ-free rearing or microbial reconstitution with CncC manipulation to establish pathway dependency. Accordingly, potential confounding effects derived from antibiotic treatment, diet, developmental stage, tissue type, host genotype and infection status must be rigorously controlled before this association can be confirmed as a general mechanism in insects.

#### 3.3.3. Regulation of Reproduction and Aging

Elevated CncC expression in the reproductive tissues of *Drosophila* points toward a functional connection with reproductive control [97]. In female *Aedes aegypti*, ovarian development is influenced by CncC-mediated regulation of the vitellogenin gene *Vg*; RNAi-mediated knockdown of *CncC* in female mosquitoes suppresses *Vg* transcript levels and compromises ovarian growth [6]. A contrasting outcome is observed in *T. castaneum*, where deltamethrin treatment induces AMPK, leading to CncC activation; activated CncC downregulates *Vg*-related gene expression and inhibits reproductive function, thereby mediating a trade-off between detoxification and reproduction [83]. In field populations of insecticide-resistant *Bemisia tabaci*, CncC exhibits constitutive activation and functions as a negative regulator of oogenesis-associated genes, which ultimately leads to abnormal ovarian development and reduced female fecundity. RNAi-mediated knockdown of *CncC* in these resistant whiteflies can upregulate the expression of the aforementioned oogenesis-related genes [98]. The discrepancies across the aforementioned observations indicate that the regulatory contribution of CncC to insect reproduction differs between species, which is likely a reflection of taxon-specific evolutionary trajectories.

**Figure 5:**
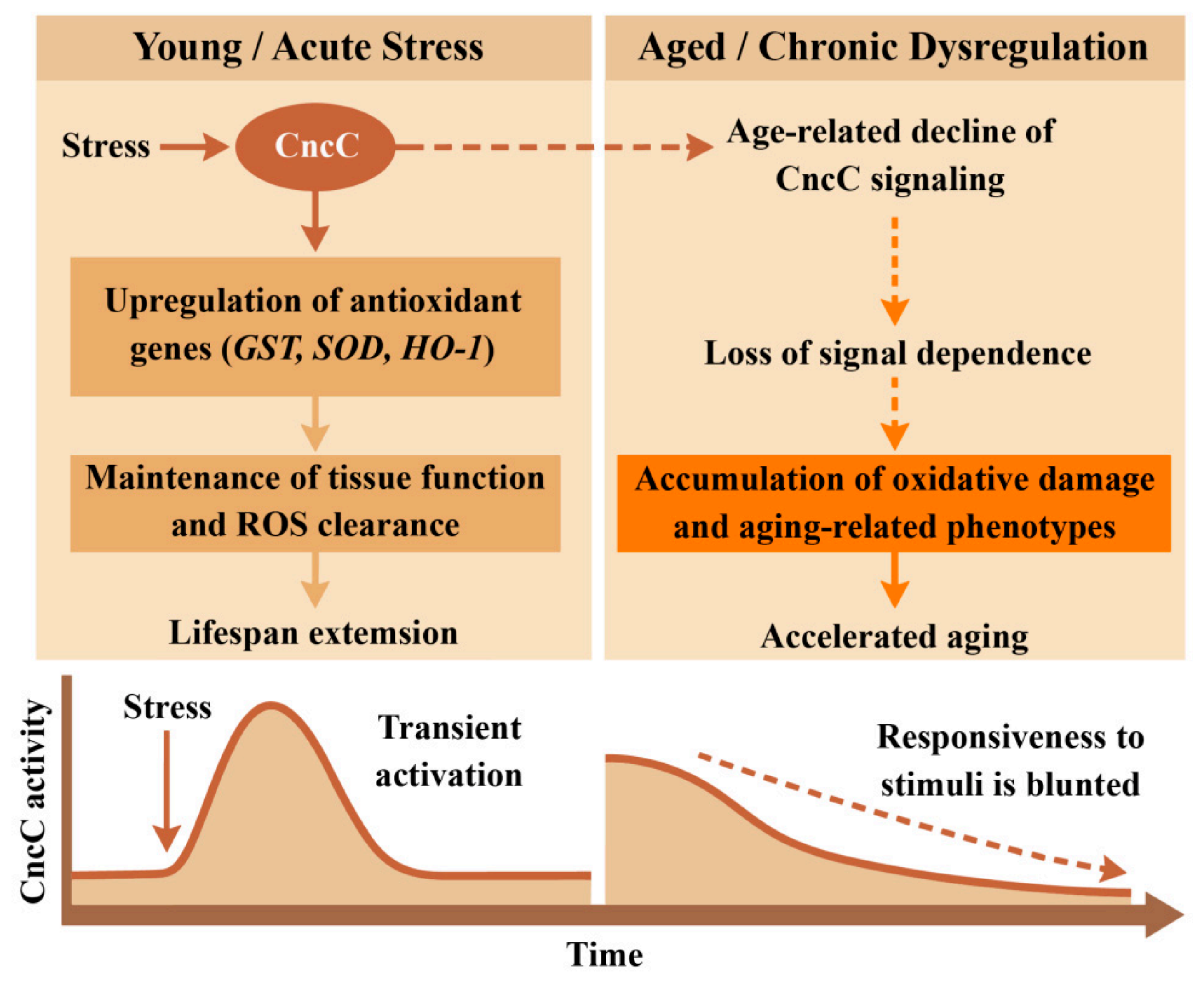
A dual-role model of CncC signaling in the regulation of insect aging. Under young or acute stress conditions (**left**), stress stimuli activate CncC, which subsequently upregulates the expression of a battery of antioxidant genes (such as *GST*, *SOD* and *HO-1*), thereby promoting reactive oxygen species (ROS) clearance and maintaining tissue function, ultimately leading to lifespan extension. In contrast, with advancing age (**right**), CncC signaling undergoes an age-related decline, characterized by blunted responsiveness to upstream stimuli (loss of signal dependence). This decline results in the accumulation of oxidative damage and the manifestation of aging-related phenotypes, ultimately accelerating the aging process. Arrow head: activation; Solid line: direct effect; Dashed line: indirect effect.

CncC activity is also intimately linked to the aging process (Figure 5). Through the activation of antioxidant and cytoprotective genes, CncC helps preserve tissue function under acute stress, an effect that can translate into extended lifespan [7]. However, excessive or dysregulated CncC signaling can exert counterproductive biological effects; constitutive and sustained activation of *CncC* in *Drosophila* induces dysregulation of the insulin/insulin-like growth factor signaling pathway, leads to the depletion of energy reserves in fly tissues and accelerates the aging process [99]. With the progression of aging, the regulatory responsiveness of the CncC pathway gradually attenuates, which is manifested as decreased sensitivity to upstream stimulation [41]. This functional decline is primarily induced by age-associated chromatin alterations, which block the binding of CncC to the promoter regions of downstream target genes. Consequently, signal-dependent transcriptional activation is abrogated, ultimately giving rise to aging-related phenotypes. Overexpression of *Maf* in aged *Drosophila* can enhance the chromatin accessibility of CncC and maintain the responsiveness of downstream genes, thereby exerting a significant anti-aging effect [7,41]. Viewed through this lens, CncC exerts a dual influence on insect aging. Its proper function hinges on transient, stress-inducible activation rather than persistent high-level expression, and the age-dependent loss of signaling responsiveness within this pathway stands as a key driver of the aging process [41,100,101].

## 4. Applied Research on CncC in Insects

The central involvement of CncC in detoxification metabolism, developmental control, and immune defense has spurred a growing body of application-oriented research in recent years. These efforts have largely coalesced around three principal directions, each explored in turn below.

### 4.1. Analysis and Management of Pest Insecticide Resistance

#### 4.1.1. CncC as a Candidate Regulator of Metabolic Resistance

Insecticide resistance remains a formidable challenge for global agriculture and public health, with metabolic resistance representing the predominant mechanism in many pest species [102,103]. The preceding sections have highlighted that CncC activation is a recurrent molecular signature of metabolic resistance across a broad range of pest species (Figure 5). However, current evidence merely supports the existence of an association between CncC and metabolic resistance. It has not yet been conclusively demonstrated that CncC acts as the dominant field-relevant determinant of metabolic resistance in most pest populations. A recent critical review published by Amezian and Van Leeuwen [53] systematically evaluated the available empirical evidence and noted that, while the CncC pathway is undoubtedly capable of conferring resistance under laboratory selection conditions and in certain field-derived strains, its prevalence as a primary field-relevant resistance determinant has not been conclusively verified in the majority of arthropod pest species. The key limitations of current research identified in the review include the widespread reliance on laboratory-selected strains and the scarcity of functional validation via reverse genetics conducted in field populations. Notwithstanding the aforementioned limitations, the functional relevance of CncC verified in laboratory-validated models has driven the development of candidate intervention strategies targeting CncC-regulated detoxification processes. Accordingly, it is necessary to categorize evidence derived from laboratory-selected strains and field-collected populations based on whether the evidence supports a mechanistic linkage, a correlative association, or a causal contribution that is relevant to field settings. Ultimately, CncC-associated detoxification should be integrated into the broader analytical framework of insecticide resistance mechanisms, alongside target-site mutations, reduced cuticular penetration, toxin sequestration, enhanced xenobiotic excretion, adaptive behavioral alterations, and resistance contributions mediated by symbiotic microorganisms.

#### 4.1.2. RNAi-Based Targeted Strategies

**Figure 6:**
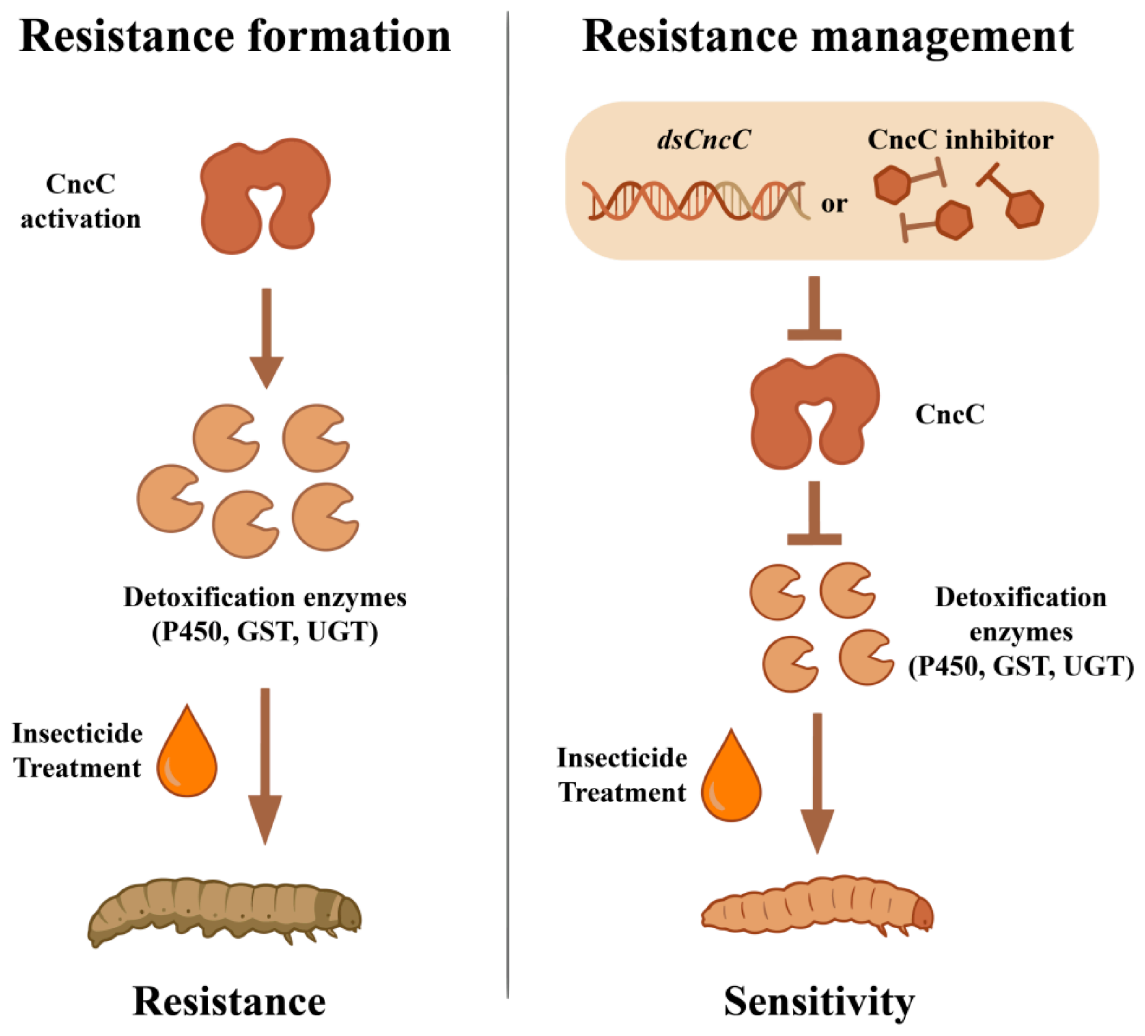
Schematic diagram of insecticide resistance formation and management based on CncC-targeted strategies. The left pathway shows that under insecticide selection pressure, constitutive activation of CncC occurs in insects, driving the overexpression of downstream detoxification enzymes (such as *P450*, *GST* and *UGT*). This enhances the detoxification capacity toward insecticides, ultimately leading to the development of resistance. The right pathway shows that downregulating CncC activity via *CncC*-targeting dsRNA or small-molecule inhibitors suppresses its transcriptional regulation of downstream detoxification enzyme genes, thereby restoring insecticide susceptibility in resistant pests. The mechanisms illustrated in this figure have been confirmed by existing experimental evidence. Arrow head: activation; Bar head: inhibiton.

RNAi-mediated silencing of *CncC* has been shown to reduce resistance to several insecticides and to produce synergistic effects when combined with conventional chemical insecticides in laboratory bioassays (Figure 6) [9,74]. Some representative studies demonstrated that microinjection of *CncC*-targeting dsRNA followed by insecticide treatment significantly increased insecticidal activity against resistant larvae [104,105]. Nevertheless, the field application of RNAi-based strategies is currently constrained by delivery barriers, including dsRNA instability, insufficient cellular uptake, and interspecific heterogeneity in gene knockdown efficiency. Nanotechnology-mediated delivery platforms are therefore being explored as promising laboratory-stage solutions.

A notable example is a hydrophilic and lipophilic diblock polymer (HLDP)-based co-delivery nanoplatform that simultaneously delivers *CncC*-targeting dsRNA and imidacloprid (IMI) to *Myzus persicae* nymphs via microinjection and feeding [106]. This study provided functional evidence for the role of CncC in metabolic resistance and showed that the self-assembled nano-delivery system can inhibit the expression of *CncC* while releasing insecticidal components, thereby reducing metabolic resistance under laboratory conditions. Similar co-delivery nanoplatforms have also been reported to deliver *CncC*-targeting dsRNA and insecticides to *S. frugiperda* and *Megalurothrix usitatus*, inhibiting *CncC* expression and enhancing insecticidal efficacy [107,108].

In a separate study targeting mite pests, which are taxonomically distinct from insects but retain a conserved CncC-ARE pathway, pH-responsive chitosan quaternary ammonium salt (HACCs) nanocomplexes enabled co-delivery of dsRNA and cyenopyrafen (Cpf) for the control of *Tetranychus urticae* and *Tetranychus truncatus* [109]. The Cpf@CHC-dsRNA/HACCs nanocomplex efficiently silenced multiple target genes, including *CncC*, *chitin synthase* (*CHS*) and *HR96*, and significantly increased mite susceptibility to Cpf. This study provides proof-of-concept that CncC-targeted co-delivery may be extendable across taxa, although direct validation in insect pests is still needed. It also highlights the potential of multi-target gene silencing for sustainable resistance management.

Although these laboratory results are encouraging, field application of CncC-targeting RNAi remains restricted by multiple critical challenges, including dsRNA degradation, ultraviolet radiation damage, rainfall erosion, inconsistent oral uptake efficiency, species-specific variation in RNAi efficacy, and insufficient delivery to target tissues. In addition, the potential impacts on non-target organisms, such as pollinators, parasitoids and predatory species, require rigorous assessment with respect to sequence specificity and realistic environmental exposure. Moreover, nanoparticle-based delivery systems remain at an early validation stage, and improvements achieved under laboratory conditions should not be interpreted as evidence that the major bottlenecks restricting field application have been fully addressed.

#### 4.1.3. Small-Molecule Inhibitor Approaches

An alternative tactic involves the deployment of small-molecule inhibitors of CncC to disrupt the transcriptional activation of resistance genes, thereby restoring pest susceptibility (Figure 6). The hypothesis that CncC inhibitors can be applied to pest control or insecticide resistance management was initially proposed in early mechanistic studies by Misra et al. [47] and Gaddelapati et al. [69]. However, for a long time thereafter, research focusing on the application of CncC inhibitors in pest management had remained extremely limited. Recently, Hu et al. [110] established a cell-based screening platform and identified sofalcone as a candidate small-molecule CncC inhibitor. A series of in vitro and in vivo experiments indicated that sofalcone can functionally inhibit the CncC pathway. However, direct binding between sofalcone and CncC has not yet been validated, and potential effects on Keap1, upstream signaling, or other components of this pathway cannot be excluded. Functionally, sofalcone suppresses CncC-mediated transcriptional activation of detoxification genes and downregulates their expression, significantly increasing insecticide susceptibility in *S. exigua*. On the basis of the above findings, Hu et al. proposed that CncC inhibitors may serve as broad-spectrum synergists to overcome insecticide resistance in pest populations. Nevertheless, the cross-species activity of sofalcone, its effects on mammalian Nrf2 signaling, its toxicity to beneficial insects and vertebrates, its environmental stability, and its impacts on development and immune function remain largely uncharacterized.

Compared with RNA interference (RNAi), small-molecule CncC inhibitors have multiple theoretical advantages. They can be formulated into conventional sprayable insecticide preparations. Their activity does depend on the endogenous RNAi machinery of target species; because the CncC-DNA interaction interface is highly evolutionarily conserved [37], they may have a broader cross-species activity spectrum. However, the screening and development of highly specific inhibitors that avoid adverse effects on non-target organisms, including beneficial insects and mammals, remain a major challenge in medicinal chemistry. Although the discovery of sofalcone as a candidate resistance reversal agent represents an important proof of concep [110], further optimization of potency, selectivity, and field stability is still required before such compounds can be applied in practical insecticide resistance management programs.

### 4.2. Development of Bioreactors Based on CncC Regulation

The CncC-ARE signaling axis is capable of sensing inducer signals and initiating the expression of downstream target genes [111,112]. Multiple low-toxicity compounds, including curcumin and sulforaphane, have been verified to act as effective CncC activators, which upregulate the expression level of target genes via this signaling pathway [113,114]. The silkworm (*B. mori*) has been widely recognized as an economically important insect and a classic model bioreactor system [115,116], and transgenic technologies have enabled efficient secretion of exogenous recombinant proteins from the silkworm silk gland [117,118]. For instance, existing research has achieved high-level expression of recombinant human type III collagen (rhCOLIII) in silkworm cocoons. Notably, this work utilized the fibroin heavy chain (FibH) expression system rather than the CncC-ARE pathway to achieve posterior silk gland-specific expression, with an obtained yield of 7.8 mg/g cocoon shell weight [119].

To date, no CncC-ARE-based inducible silkworm bioreactor has been reported. Nonetheless, existing studies have confirmed high structural and mechanistic homology between the insect CncC system and its mammalian ortholog Nrf2 [37], indicating that this inducible expression platform could potentially be constructed in silkworms. In the proposed design, the CncC-ARE pathway can drive the inducer-dependent precise expression of exogenous genes, including those encoding pharmaceutical proteins and vaccine antigens in target tissues. A potential advantage of this system is that it may reduce the metabolic burden caused by constitutive overexpression, thereby possibly improving the operational safety and overall controllability of the bioreactor. However, this benefit needs experimental verification. A number of limitations of the proposed system also require consideration, including basal ARE activity, tissue specificity, inducer toxicity, induction kinetics, expression strength, unintended activation induced by environmental stress, as well as potential impacts on silk production.

### 4.3. Environmental Risk Assessment and Biomarker Applications

The responsiveness of the CncC pathway to environmental chemicals (such as insecticides and heavy metals) has made CncC-related readouts a promising biomarker for monitoring the ecological risks of environmental pollutants to non-target insects (such as honeybees and natural enemy insects) [120,121,122,123]. In environmental toxicology studies, measuring changes in CncC and its downstream genes in honeybees or ladybeetles may provide a rapid indication of the effects of sublethal pesticide exposure on oxidative stress status and physiological performance [124]. Compared with a mortality-based endpoint, biomarker-based assessments can offer greater sensitivity and earlier warning of sublethal effects under certain conditions [125,126]. However, the transcript abundance of *CncC*, the protein abundance of CncC or its nuclear localization, as well as the expression of downstream target genes or corresponding enzyme activity, reflect different dimensions of pathway activation status and do not necessarily exhibit consistent changes. Additionally, the effects of age, sex, tissue type, developmental stage, exposure duration, and environmentally relevant exposure concentrations all need to be taken into account. Until sufficient further validation is completed, CncC-related readouts are more appropriately defined as candidate early-warning biomarkers or potential components of a multi-biomarker panel. A promising direction for future research is to determine whether CncC activation signatures can distinguish general oxidative stress from exposure to specific classes of toxicants, which would enhance the diagnostic value of CncC-based biomarkers in environmental contamination assessment.

## 5. Conclusions and Future Perspectives

The transcription factor CncC has emerged as a central orchestrator of cytoprotective responses in insects, yet its functional repertoire now extends well beyond the classical domains of antioxidant defense and xenobiotic detoxification. Contemporary investigations have uncovered critical roles for CncC in processes as diverse as organismal development, immune regulation, reproductive physiology, and the acquisition of insecticide resistance. Notably, CncC has evolved a regulatory network that interacts with the ecdysone signaling pathway and the gut microbiome, reflecting its unique contribution to insect metamorphosis and ecological adaptability. The field is currently undergoing a conceptual transition. The focus is shifting from the characterization of individual gene functions toward a more integrative understanding of network regulation, cross-species divergence, and the translation of fundamental insights into practical applications.

Despite this progress, several key scientific questions remain to be elucidated. First, the question of how CncC achieves differential activation of target genes in distinct tissues, and whether tissue-enriched co-regulatory factors contribute to this specificity are matters that warrant systematic investigation. Future investigations utilizing single-cell transcriptomic technologies are critically required to deconstruct the tissue-specific regulatory mechanisms underlying CncC activation. Second, although CncC activation contributes to enhanced insecticide resistance, long-term or excessive activation is often accompanied by fitness costs such as developmental delay and reduced fecundity. The underlying mechanisms of metabolic trade-offs currently remain unelucidated, and characterization of these constraints is indispensable for predicting the evolutionary dynamics of resistance in field populations. Third, although RNAi and small-molecule inhibitors targeting CncC have shown significant efficacy in laboratory-based research, multiple critical challenges still exist for their field applications, which include realizing efficient delivery systems, lowering development and application costs, and eliminating off-target effects on non-target organisms. Fourth, the core scientific question of whether alteration of the CncC/Keap1 pathway constitutes the dominant resistance mechanism in the majority of field pest populations remains to be rigorously validated in diverse field populations using a combination of reverse genetic approaches and population genomic analyses. Answering this question is indispensable for confirming the generalized applicability of CncC-targeted resistance management strategies across different pests. In addition, CncC functions within an expanded regulatory network, and its interactions with other transcription factors and signaling pathways such as small Maf, HR96 (hormone receptor 96), AhR-related signaling, FOXO (forkhead box class O), HNF4 (hepatocyte nuclear factor 4), ecdysone receptor signaling, JNK (c-Jun N-terminal kinase), and TOR (target of rapamycin), as well as chromatin regulation and non-coding RNAs, are currently insufficiently characterized and require targeted follow-up investigation.

Moving forward, the integration of single-cell sequencing, gene editing, structural biology, and synthetic biology technologies will enable comprehensive dissection of the multiple functions of CncC in insect life activities, from the molecular level to the ecosystem level. These studies will not only deepen our understanding of insect environmental adaptability but also provide key targets and theoretical support for the development of novel, efficient, green and environmentally sustainable pest management strategies.

## Figures and Tables

**Table 1:** Study on CncC in insect detoxification metabolism and resistance.

Insect Species	System	Verification Technology	Downstream Genes	Observed Phenotypes	Types of Toxic Substances	References
*Tribolium castaneum*	Larvae	Induce, RNAi	*CYP4Q3*, *CYP4Q5*, *CYP4Q7*	Eugenol tolerance	Phytochemical	[55]
*Spodoptera litura*	Larvae	Induce	*GST*	Indole-3-methanol (I3C) tolerance	Phytochemical	[56]
*Helicoverpa armigera*	Larvae	Induce, RNAi	*CYP321A1*	Flavone tolerance	Phytochemical	[58]
*Spodoptera litura*	Larvae/Cells	Induce, RNAi, Dual-luciferase reporter	*UGT33B28*, *UGT33V9-2*	Xanthotoxin tolerance	Phytochemical	[59]
*Spodoptera litura*	Larvae/Cells	Induce, RNAi, Dual-luciferase reporter	*CYP321A*	Flavone, Xanthotoxin, Curcumin, λ-Cyhalothrin tolerance	Phytochemical, Insecticide	[60]
*Micromelalopha troglodyta*	Cells	Induce, Dual-luciferase reporter	*GSTt1*	Tannic acid and quercetin induce gene expression	Phytochemical	[61]
*Aphis gossypii*	Larvae/Cells	Induce, RNAi, Dual-luciferase reporter	*CYP6DA2*	Gossypol tolerance	Phytochemical	[62]
*Dendroctonus armandi*	Larvae	Induce, RNAi, Agonist	*CYP4BQ1*	(+)-α-pinene tolerance	Phytochemical	[64]
*Spodoptera litura*	Larvae/Cells	Induce, RNAi, Dual-luciferase reporter, EMSA	*GSTe1*	I3C tolerance	Phytochemical	[66]
*Bradysia odoriphaga*	Larvae	Induce, RNAi, Agonist	*CYP3828A1*, *CYP6FV21*	Clothianidin resistance	Insecticide	[67]
*Leptinotarsa decemlineata*	Larvae/Cells	Induce, RNAi, Dual-luciferase reporter	*CYP9Z25*	Imidacloprid resistance	Insecticide	[68]
*Leptinotarsa decemlineata*	Larvae	Induce, RNAi	*ABCH278B*	Imidacloprid resistance	Insecticide	[69]
*Spodoptera litura*	Larvae	Induce, RNAi, Agonist	*CYP6AB12*	λ-Cyhalothrin tolerance	Insecticide	[70]
*Spodoptera litura*	Larvae	Induce, RNAi	*CYP367A1*, *CYP367B1*, *CYP341B21*, *CYP340L2*, *CXE1*	Indoxacarb resistance	Insecticide	[71]
*Spodoptera litura*	Larvae	Induce	*UGT386B2*	Imidacloprid resistance	Insecticide	[72]
*Nilaparvata lugens*	Larvae	Induce	*CYP6ER1*	Imidacloprid resistance	Insecticide	[73]
*Spodoptera frugiperda*	Larvae/Cells	Induce, RNAi, Dual-luciferase reporter, ChIP	*CYP321A8*, *GSTe9*, *UGT33B13*	Deltamethrin resistance	Insecticide	[9]
*Drosophila melanogaster*	Larvae	Induce, RNAi	*CYP6a8*, *CYP6a2*	Dichlorodiphenyltrichloroethane (DDT) resistance	Insecticide	[47]
*Spodoptera exigua*	Larvae/Cells	Induce, Dual-luciferase reporter	*CYP321A8*	Chlorpyrifos, cypermethrin, deltamethrin resistance	Insecticide	[75]
*Aphis gossypii*	Larvae/Cells	Induce, RNAi, dual-luciferase reporter, yeast one-hybrid assays	*CYP4CJ2*	Cycloxaprid tolerance	Insecticide	[76]
*Lymantria dispar*	Larvae	Induce, RNAi	*CYP6B7v1*, *CYP321A7*, *CYP4S4v1*	Emamectin benzoate tolerance	Insecticide	[77]
*Spodoptera exigua*	Larvae/Cells	Induce, Dual-luciferase reporter	*CYP321A16*, *CYP332A1*	Chlorpyrifos resistance	Insecticide	[78]
*Tribolium castaneum*	Larvae	RNAi	*CYP4G7*, *CYP4G14*, *GST-1*	Pyrethroid resistance	Insecticide	[3]

## Data Availability

No new data were created or analyzed in this study. Data sharing is not applicable to this article.
